# To *B* or not to *B*: a question of resolution?

**DOI:** 10.1107/S0907444911028320

**Published:** 2012-03-16

**Authors:** Ethan A. Merritt

**Affiliations:** a Department of Biochemistry, University of Washington, Seattle, WA 98195-7742, USA

**Keywords:** atomic displacements, *B* factors, TLS models, model parameters

## Abstract

In choosing and refining any crystallographic structural model, there is tension between the desire to extract the most detailed information possible and the necessity to describe no more than what is justified by the observed data. A more complex model is not necessarily a better model. Thus, it is important to validate the choice of parameters as well as validating their refined values. One recurring task is to choose the best model for describing the displacement of each atom about its mean position. At atomic resolution one has the option of devoting six model parameters (a ‘thermal ellipsoid’) to describe the displacement of each atom. At medium resolution one typically devotes at most one model parameter per atom to describe the same thing (a ‘*B* factor’). At very low resolution one cannot justify the use of even one parameter per atom. Furthermore, this aspect of the structure may be described better by an explicit model of bulk displacements, the most common of which is the translation/libration/screw (TLS) formalism, rather than by assigning some number of para­meters to each atom individually. One can sidestep this choice between atomic displacement parameters and TLS descriptions by including both treatments in the same model, but this is not always statistically justifiable. The choice of which treatment is best for a particular structure refinement at a particular resolution can be guided by general considerations of the ratio of model parameters to the number of observations and by specific statistics such as the Hamilton *R*-­factor ratio test.

## Abstract 2

A simple rule of thumb based on resolution is not adequate to identify the best treatment of atomic displacements in macromolecular structural models. The choice to use isotropic *B* factors, anisotropic *B* factors, TLS models or some combination of the three should be validated through statistical analysis of the model refinement.

## 1. Introduction   

Since at least the time of the 14th-century logician William of Occam, scientific models have been scrutinized for the possible flaw of being overly complex. A succinct modern formulation of Occam’s ‘razor’ is the admonition by Albert Einstein that ‘everything should be made as simple as possible, but not simpler’. Our confidence in a complicated model with many adjustable parameters is weakened if it is supported by only a small number of data points. Conversely, we assign only weak utility to a simplistic model that fails to explain obvious patterns in a very rich set of observations. Unfortunately, it is not always a straightforward task to decide whether a model is too complex or too simple.

**Figure 1:**
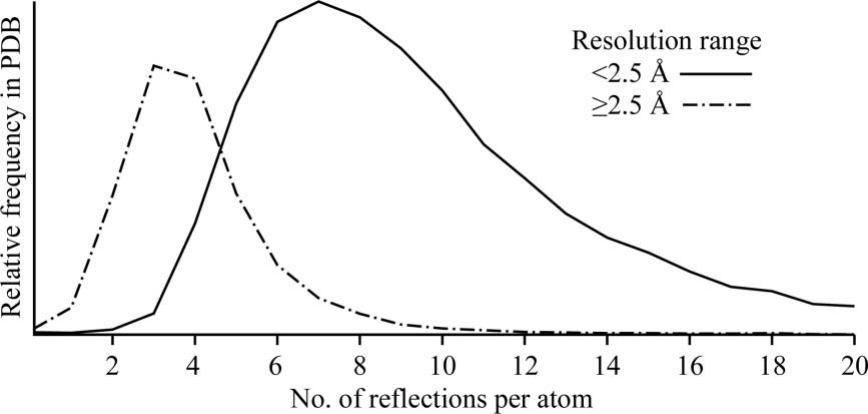
Number of reflections per atom for all X-ray crystal structural models in the PDB (February 2011). Note that this number depends on the solvent fraction of the crystal. A large number of reflections per atom usually corresponds to a structure refined against very high resolution data, but it may also indicate a structural model that describes only one copy of a molecule present in multiple copies related by explicit noncrystallographic symmetry, *e.g.* one subunit of an icosahedral virus capsid.

Applying Occam’s razor to structural models in biological crystallography is rarely straightforward. Although the number of observations is large, the number of parameters required to describe a biological macromolecule is also very large. If the diffraction measurements are limited to 2 Å resolution then there are typically about eight intensity measurements available for each non-H atom in a protein crystal structure. If one models each atom as having a position [*x, y, z*] and a thermal parameter *B*_iso_, this corresponds to ∼2 observations per model parameter (Fig. 1 ▶). As Lyle Jensen observed in earlier days of macromolecular crystallography, ‘The problem is overdetermined and there is no *in principle* reason why refinement should not be possible’ (Jensen, 1974 ▶). The number of observations falls as the cube of the resolution, however, so that the problem ceases to be overdetermined at lower resolutions. Thus, in practice, model refinement is possible only if the experimental observations are supplemented with restraints on the model’s geometric and other properties. In a typical protein refinement at less than atomic resolution the number of restraints can be much larger than the number of observations. Sufficiently strong restraints can force the model to become numerically well behaved during refinement. The restraints mitigate, but do not remove, the concern that inclusion of unjustified parameters in the model being refined can degrade the model quality through overfitting. A simple model with fewer restraints may still be better than a more complex highly restrained model, particularly when low resolution limits the observation-to-parameter ratio.

How, then, can one decide whether a model has been sufficiently restrained, whether it has been overfitted and whether it is too complex? I will first consider some general approaches and then examine a series of examples in which there is a choice between a simpler model and a more complex model. In all of these examples the difference in model complexity arises from different parameterization of the description of atomic displacements. The simplest such treatment is to assign only an overall description *U*_overall_ that applies equally to all atoms. This results in a model that contains three parameters [*x*, *y*, *z*] for each atom plus one to six global parameters depending on whether *U*_overall_ is isotropic or anisotropic. Thus, for an *N*-atom structure the simplest model contains (3*N* + 1) parameters. The most complex model that we will consider is a treatment that assigns each atom an individual 3 × 3 symmetric tensor *U^ij^* describing a thermal ellipsoid, which yields a total of 9*N* model parameters. Between these two extremes are hybrid models that contain some combination of individual per-atom isotropic terms *B*_iso_ and translation/libration/screw (TLS) group descriptions of bulk displacement (Schomaker & Trueblood, 1968 ▶; Winn *et al.*, 2001 ▶; Painter & Merritt, 2006 ▶).

Until recently, the issue of how to model atomic displacement was often reduced to a rough rule of thumb that at very high resolution one should model anisotropy of individual atoms using a six-parameter thermal ellipsoid, at very low resolution one should assign a shared *B* factor to groups of atoms and for everything in between one should refine an isotropic *B* factor for each atom. Even aside from the vagueness of where to draw the boundaries for ‘very high’ and ‘very low’ resolution, the introduction of TLS as an alternative description of atomic displacement has made this rule of thumb obsolete. Unfortunately, it sometimes seems to have been replaced by an assumption that the best treatment at all resolutions is to include both individual isotropic *B* factors and some number of TLS groups. I advocate that rather than following any such rule of thumb, the best treatment of displacements and anisotropy should be validated for each structure based on the experimental data and refinement statistics.

## 2. Validating a model: has something gone wrong?   

In validating an existing structural model we are confirming that it does not conflict with the experimental data and equally that it does not conflict with prior knowledge. Validation is essential to assure confidence in the scientific conclusions drawn on the basis of the model. Comprehensive overviews of crystallographic model validation may be found elsewhere (Kleywegt, 2000 ▶, 2009 ▶; Chen *et al.*, 2010 ▶). Here, we will only touch briefly upon issues related to validation of *B* factors or other descriptions of atomic displacement.

### 2.1. Agreement of the model with the experimental data   

The agreement of the model with the experimental data is conventionally quantified as a crystallographic residual *R*, which may be calculated using intensities (*I*) or structure factors (*F*). Several variants of *R* are in common use. The conventional unweighted residual *R* calculated for *F* is given by
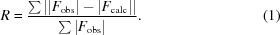

*R* is closely related to the target function being minimized during refinement. Therefore, if the same observations con­tributing to refinement are used to calculate *R*, its value will normally decrease as a consequence of refinement. In order to test for overfitting, a fraction of the reflections may be omitted from the target function during refinement. Two residuals are then calculated. The reflections used in refinement are used to calculate *R*_work_, and the remaining reflections omitted from refinement are used to calculate a corresponding residual *R*_free_. If *R*_free_ does not decrease in parallel with *R*_work_ as a result of refinement, this is an indication of overfitting (Brünger, 1992 ▶, 1997 ▶; Tickle *et al.*, 1998 ▶, 2000 ▶). It is important to note that in this context *R*_free_ is being used to validate choices affecting the progress of a refinement, for example the strength of restraint weights, rather than to validate the selection of a model to be refined.

A related quantity, the use of which will be explored below, is Hamilton’s generalized residual *R*_G_,
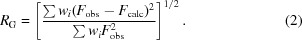
The virtue of Hamilton’s residual is that significance tests involving *R*_G_ can be directly related to the standard statistical *F* test (Hamilton, 1965 ▶).

### 2.2. Agreement of the model with prior knowledge   

Most validation tests assess agreement of the model with prior knowledge. These tests encompass everything from detecting local problems such as a single poorly modeled residue to global issues such as implied inconsistency with the known biological properties of the molecule. For example, there is much prior knowledge about bond lengths and angles in organic molecules and about the joint distribution of the paired torsion angles [ϕ, ψ] at each peptide linkage in a protein. Significant deviation from these expectations for a particular residue may indicate a local problem, reducing our confidence in local features of the model, without necessarily implying that the overall model is poor.

Analogous prior expectations can be applied to detect local problems in atomic displacement parameters (ADPs). The tensor *U^ij^* describing anisotropic displacement of a particular atom, whether refined directly or derived from inclusion in a TLS group, should be positive definite. Bonded atoms are expected to exhibit similar atomic vibrations; in particular, the vibrational components along their mutual bond are expected to be equal (Hirshfeld, 1976 ▶; Rosenfield *et al.*, 1978 ▶). The overall distribution of anisotropy for atoms in a protein crystal structure is expected to be approximately Gaussian, with a mean axial ratio in the range 0.45–0.55 (Merritt, 1999*a* ▶; Zucker *et al.*, 2010 ▶). If atomic displacements within a protein chain are described by segmenting the chain into multiple TLS groups, then the vibration of atoms at the junction of two adjoining TLS groups is expected to be described consistently by both sets of TLS parameters (Zucker *et al.*, 2010 ▶). One resource that provides validation tests based on agreement with prior expectations for these various properties of displacement parameters is the *PARVATI* server http://www.bmsc.washington.edu/parvati.

### 2.3. A caution about the meaning of *B* factors   

There is a possible problem in validating *B* factors. Before we can identify prior expectations for their distribution, we must first establish the physical meaning of individual values. The IUCr defines both isotropic and anisotropic ADPs as representing ‘atomic motion and possible static displacive disorder’ (Trueblood *et al.*, 1996 ▶). Under this interpretation the [*x*, *y*, *z*] coordinates of an atom represent its true mean position, and the ADP values represent displacement about this mean position. Because we understand this displacement as arising from physical vibration, we can establish an expected distribution of ADP values based on models of physically reasonable modes of vibration. We expect that vibrational modes involving multiple atoms will lead to correlated displacement of those atoms and hence to correlated ADP values.

However, some programs also use or allow large *B* values to represent general uncertainty that a portion of the structure has been correctly modeled. Under this interpretation the nominal [*x*, *y*, *z*] coordinates may not be correct at all, and the ‘*B* value’ is a measure of relative confidence rather than displacement about some mean position. Although it could be argued that being somewhere else entirely is an example of ‘displacive disorder’ allowed by the IUCr definition, such interpretation is of little use in establishing prior expectations or validation criteria. From this perspective, general uncertainty and the known presence of multiple possible locations of the atom or group in question are both represented better by occupancy <1 rather than by an arbitrarily large *B* factor. This distinction is particularly important if the *B* values are used to determine, refine or validate the assignment of TLS groups.

## 3. The other half of validation: is this the right model?   

While validation of the stereochemistry and other physical properties of a model after refinement is essential, it is not the end of the story. It neither asks nor answers the question ‘was this the best model to refine?’. In particular, it does not address the question of whether a simpler model would suffice. I have already noted that a more complex model is expected to yield a better residual *R* after refinement, and that a failure to reduce *R*_free_ in parallel is an indicator for overfitting. However, even if the more complex model yields both lower *R* and lower *R*_free_, we can still ask whether this improvement is statistically significant.

### 3.1. Hamilton *R*-value ratio test   

One approach is to compare the residuals obtained experimentally for the new structure with either empirical or theoretical expectations for the conventional *R* and *R*_free_ obtained for a model of this size and complexity (Kleywegt & Brünger, 1996 ▶; Tickle *et al.*, 2000 ▶). In order to derive a quantitative significance level, it is preferable to replace the con­ventional residuals with variants whose statistical properties are better defined. If one replaces the conventional residual *R* with the generalized residual *R*_G_ (equation 2), then it is possible to derive significance by consideration of the ratio of the *R* factors for the simple and the complex models (Hamilton, 1965 ▶). Hamilton’s original formulation considered the case in which a simpler model was related to a more complex model by the addition of a set of linear constraints. Furthermore, Hamilton was concerned with the typical crystallo­graphic problems of the day, for which both the number of observations and the number of parameters were small and the weighting factor *w_i_* used in refinement was the same for all reflections.

Bacchi *et al.* (1996 ▶) reformulated this approach for application to macromolecular models, where both the number of observations and the number of parameters are much larger and both the simple and complex models are refined with restraints. With a slight change in notation, we may restate the reformulated significance test as follows.

Let us define the degrees of freedom for model refinement as 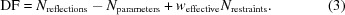
Now consider two refined models with residuals *R*_G_(1) and *R*_G_(2) and degrees of freedom DF(1) and DF(2). Let model 2 be the more complex model; by which we mean that it has more parameters and/or fewer restraints. By (3) above, the complex model has fewer degrees of freedom than the simpler model, so DF(1)/DF(2) is always greater than one. The simpler model is expected to have a higher *R* factor than the more complex model, in which case the ratio *R*_G_(1)/*R*_G_(2) will also be greater than one. However, the lower *R* factor for the more complex model indicates a significant improvement only if this ratio also satisfies
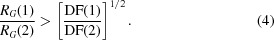
Note that the number of degrees of freedom depends on an effective restraint weight defined such that *w*_effective_ = 0 corresponds to ignoring the restraints and *w*_effective_ = 1 corresponds to treating each restraint as a full constraint analogous to adding one observation or reducing the parameter count by one parameter. Because we will be considering model pairs that differ only in their treatment of ADPs, we further sub­divide the restraints into geometric restraints present in both models and ADP restraints that may be present in only one of the two models, 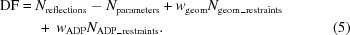



### 3.2. Limitations   

A major difficulty in applying the Hamilton *R*-factor ratio test is that the value of *w*_effective_ is in general unknown. In some cases the analysis can proceed nevertheless by evaluating (4) across the entire range of possible values for *w*_effective_ (Bacchi *et al.*, 1996 ▶). If the test for significance yields the same result when evaluated at both extreme values of *w*_effective_ then we can accordingly either accept or reject the more complex model even though the exact value of [DF(1)/DF(2)]^1/2^ remains unknown. One extreme, *w*_effective_ = 0, corresponds to unrestrained refinement. Evaluation of (4) at this extreme is straightforward. The other extreme is bounded by *w*_effective_ < 1, but 1 is a very weak upper bound that could only be reached if all restraints were independent. In practice, the restraints applied during macromolecular refinement are far from independent (there are many more restraints than there are parameters) and are assigned a fractional weight during refinement in order to balance their contribution to the overall residual. As a result, *w*_effective_ << 1.

It is possible that one could derive a good estimate for *w*_effective_ based on the deviation of the restrained parameters from their target restraint values at the end of refinement, *i.e.* the largest deviations are expected when the refinement is unrestrained (*w*_effective_ = 0) and the smallest deviations, possibly zero, are expected when the restraints are so tight that they act as constraints. However, no quantitative procedure for making such an estimate has yet been developed. Nevertheless, for the examples presented below we use this argument to set an upper bound on the possible values of *w*_geom_ and *w*_ADP_. Since a fully constrained geometric model would require no more than one constraint per coordinate, we set an upper bound on the limiting condition max(*w*_geom_) = 3 × *N*_atoms_/*N*_geom_restraints_. Similarly, a fully constrained set of isotropic ADP values would require at most one constraint per atom, so we set an upper bound on the limiting condition max(*w*_ADP_) = *N*_atoms_/*N*_ADP_restraints_. These are weak upper bounds, a fact that can be seen empirically by noting that refinement of the restrained model does not normally con­verge to a model in which the parameter values fully conform to the restraint targets as they would for true constraints.

The upper bound for *w*_ADP_ is especially weak, because all of the restraints applied to isotropic ADPs during refinement contain a multiplicative term of the form [*B*(atom *i*) − *B*(atom *j*)], where *B* is either the individual isotropic *B*_iso_ or the residual per-atom contribution to a TLS model *B*_resid_. Thus, for isotropic ADPs a fully constrained model satisfying these restraints would have equal *B* terms for all atoms. The limiting case of a model refined with max(*w*_ADP_) as defined above converges to being identical to a simpler model with a single *B*_overall_ and perhaps one or more TLS groups. Thus, we know that in practice *w*_ADP_ << max(*w*_ADP_) both because the *R* factors for the simple and complex models are different and because the refined *B* values are not, in fact, identical.

## 4. Worked examples   

### 4.1. Choices at low resolution: overall *U* ^*ij*^, individual *B*_iso_ or pure TLS   

**Figure 2:**
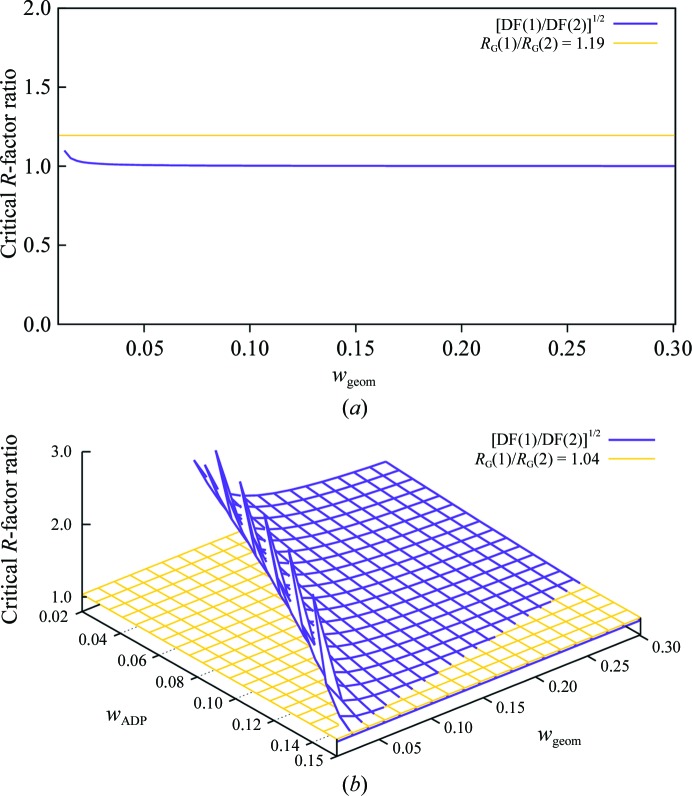
(*a*) Application of the Hamilton *R*-factor ratio test to comparison of *U*_overall_ and pure TLS models. The structure being refined is a tryptophanyl-tRNA synthetase homolog from *Entamoeba histolytica* (PDB entry 3hzr). The simple model 1 contains six ADP parameters *U* *^ij^*_overall_. The more complex model 2 contains six TLS groups for a total of 120 ADP parameters. The effective weight of the geometric restraints *w*_geom_ is unknown, so we calculate the function DF(1)/DF(2) over all possible values of *w*_geom_ and show that it is less than the observed *R*-factor ratio *R*_G_(1)/*R*_G_(2) = 1.19 everywhere in this range. The quantity *w*_ADP_ is not relevant because neither model contains ADP restraints. (*b*) Application of the Hamilton *R*-factor ratio test to comparison of pure TLS and *B*_iso_ models. In this case the pure TLS model 1 with 120 ADP parameters is the simpler model. The more complex model 2 contains 17 732 *B*_iso_ parameters, one for each atom. In this comparison both *w*_geom_ and *w*_ADP_ are needed but unknown, so DF(1)/DF(2) becomes a two-variable function depending on both. According to the Hamilton ratio test, the more complex model is justifiable only when the *R*-factor ratio *R*_G_(1)/*R*_G_(2) = 1.04 (yellow surface) is greater than DF(1)/DF(2) (purple surface). This condition holds only along the far-right edge of the plot corresponding to ADP restraint weights so tight that they approach the limiting condition of a constraint to equal *B*_iso_ for all atoms.

The number of observations available per model parameter becomes an increasing concern for lower resolution data. At 3 Å resolution, the number of available observations is insufficient to support refinement of four parameters per atom in the absence of additional restraints (Fig. 1 ▶). Depending on the individual structure being modeled, the available data may or may not justify refinement of separate ADPs for each atom even in the presence of restraints. Table 1 ▶ and Fig. 2 ▶ illustrate the use of refinement statistics to guide the choice between refining a conventional model with four parameters per atom (*x*, *y*, *z*, *B*_iso_) or a simpler model containing no per-atom displacement parameters. We chose PDB entry 3hzr (Merritt *et al.*, 2011 ▶) as a representative 3 Å resolution structure to use for this example. The 3hzr model contains three dimers in the asymmetric unit, comprising a total of 2262 protein residues with no water molecules or other nonprotein atoms.

We first consider the choice between two very simple models that contain no per-atom displacement parameters. The simpler of the two models contains six parameters *U* ^*ij*^_overall_. The slightly more complex alternative is a pure TLS model containing one TLS group to describe each protein chain for a total of 120 ADPs (six protein chains, one TLS group per chain, 20 parameters per TLS group). The more complex model yields substantially lower residuals *R* and *R*_free_ (Table 1 ▶). The corresponding Hamilton *R*-factor ratio is 0.2827/0.2366 = 1.19. Although we do not know the exact value of *w*_geom_, in this case the ratio DF(1)/DF(2) is insensitive to this unknown parameter and is strictly less than 1.19 over the entire range of possible values for *w*_geom_ (Fig. 2 ▶ *a*). Therefore, the improvement in residuals for the more complex model is significant and we choose the pure TLS model over the simpler alternative model.

We next compare the pure TLS model in turn to a more complex model with no TLS but containing one ADP, *B*_iso_, for each atom. The conventional *R* factor yielded by refinement is nearly the same for both models, but *R*_free_ is considerably higher for the more complex model (Table 1 ▶). Therefore, in this case examination of the conventional *R* factors already indicates that the more complex model is not justified. Let us see what the Hamilton *R*-factor ratio test indicates. For this test case *R*_G_(1)/*R*_G_(2) = 1.04 and the criterion in (4) could only be satisfied for values of *w*_ADP_ very near its limiting value *N*_atoms_/*N*_ADP_restraints_ (Fig. 2 ▶ *b*). However, we know that *w*_ADP_ is not near the limiting case of fully constrained *B*_iso_ values, because that would correspond to a model in which all ADP values are nearly equal. That is, the limiting case of maximal *w*_ADP_ is equivalent to the model with a single overall description *U*_overall_, which we have already considered and rejected. For values of *w*_geom_ and *w*_ADP_ away from their limiting maxima, the Hamilton test indicates rejection of the more complex model with individual *B*_iso_ parameters in favor of the simpler pure TLS model.

This set of tests does not inevitably yield the same decision (that one should use a pure TLS model) when applied to other 3 Å resolution structure refinements. Although we selected 3hzr as representative, it has at least two features that are atypical. Its solvent content is 56%, which is higher than average and results in a slightly higher number of observations per atom than most 3 Å resolution structures. This would tend to increase our expectation that the more complex *B*_iso_ model might be statistically justified. Counteracting this tendency, the structure exhibits atypically extreme overall anisotropy (*A*_mean_ = 0.30, σ_A_ = 0.15), perhaps owing to loose lattice packing. The simple TLS model allows description of this anisotropy, whereas the more complex *B*_iso_ model does not. This raises the question whether in this particular case the *B*_iso_ model is a failure not because of the larger number of parameters, but because it fails to account for anisotropy. We will next test whether a more complex hybrid model that includes both *B*_iso_ terms and TLS terms is statistically justified.

### 4.2. Hybrid models   

**Figure 3:**
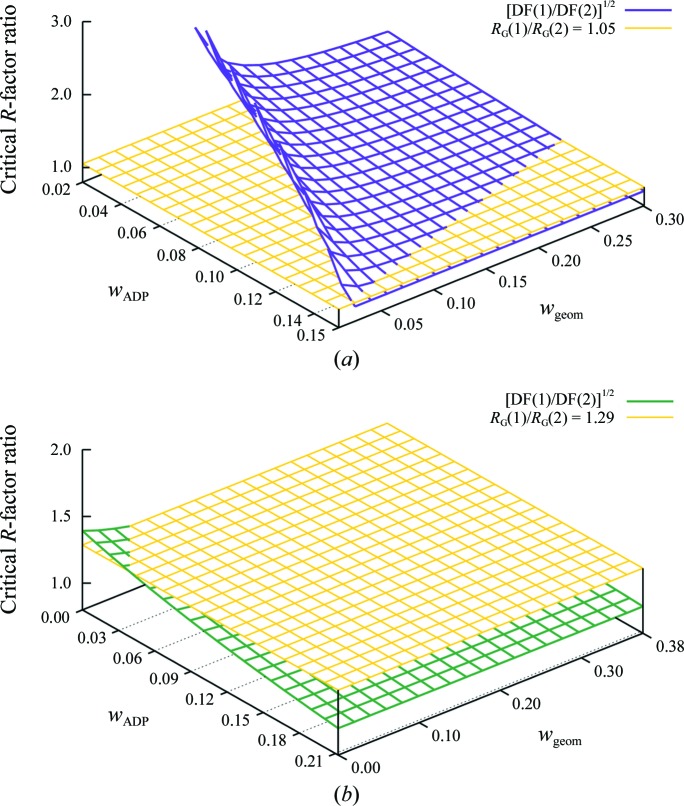
Application of the Hamilton *R*-factor ratio test to comparison of a hybrid model containing both TLS and *B*_iso_ terms to a model containing only one or the other. (*a*) Comparison of a pure TLS model to a hybrid TLS + *B*_iso_ model for the 3 Å resolution refinement of 3hzr. The acceptance surface is only slightly larger than that shown in Fig. 2 ▶(*b*). (*b*) Comparison of a pure TLS model to a hybrid TLS + *B*_iso_ model for the 2.32 Å resolution refinement of the homologous tryptophanyl-tRNA synthetase from *Campylobacter jejuni* (PDB entry 3m5w). In this case the *R*-factor ratio (yellow surface) is greater than DF(1)/DF(2) (green surface) everywhere except the bounding limit *w*_ADP_ = *w*_geom_ = 0.

It has become increasingly common to model the ADPs in a macromolecular structure using both an individual *B*_iso_ parameter for each atom and some form of TLS model to describe anisotropic displacement of those same atoms. Let us continue examination of the 3hzr refinement to evaluate the justification for such a hybrid model at the low end of the resolution range where it might be applicable (Fig. 3 ▶ *a*). The simpler model in this case is the same pure TLS model with one TLS group per chain used in Fig. 2 ▶(*b*). The more complex model in this case includes these same TLS groups and in addition contains individual *B*_iso_ terms for the protein atoms. The surfaces in Fig. 3 ▶(*a*) are remarkably similar to that in Fig. 2 ▶(*b*) and the conclusion is the same. The more complex model is statistically justified only if we believe that the *B*_iso_ parameters are so tightly restrained that they are close to functioning as constraints.

Fig. 3 ▶(*b*) shows the application of the same significance test using as a test case the structure of a homolog to 3hzr that was determined at 2.32 Å resolution (PDB entry 3m5w; Center for Structural Genomics of Infectious Diseases, unpublished work). The refinement statistics for 3m5w are given in Table 2 ▶. In contrast to the case of 3hzr, the hybrid model for 3m5w is superior to the pure TLS model with one TLS group per chain for all possible restraint weights except the unrealistic set *w*_ADP_ = *w*_geom_ ≃ 0 corresponding to unrestrained refinement.

It may seem natural to use an analogous significance test to determine whether or not it is justified to add a TLS description to a model that has already been refined with individual *B*_iso_ parameters. However, the Hamilton *R*-factor ratio is only a weak test for this purpose because the change in the overall number of parameters is very small. That is, there are typically already thousands of ADP parameters; adding 20 more for each TLS group is a very small incremental change. For a structure with thousands of atoms per chain, associating an additional 20 TLS parameters with each chain will yield a test criterion [DF(1)/DF(2)]^1/2^ on the order of 1.001. Thus, according to the *R*-factor ratio criterion, the addition of TLS can be justified by any marginal improvement in the residuals. In the particular case of 3m5w, the simple TLS model describing each protein chain by a single TLS group yields only a slight improvement in the conventional *R* and *R*_free_ (Table 2 ▶) and the corresponding Hamilton *R*-factor ratio is only *R*_G_(1)/*R*_G_(2) = 1.01. Nevertheless, this is larger than the test criterion for all possible values of the effective restraint weights, justifying acceptance of the hybrid model.

### 4.3. Hybrid models at high resolution   

**Figure 4:**
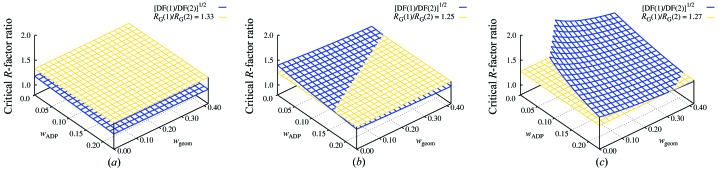
Application of the Hamilton *R*-factor ratio test to validate use of a fully anisotropic model for carbonic anhydrase at various resolutions. The simpler model is a hybrid model that contains 16 TLS groups in addition to *B*_iso_ terms for each atom. The more complex model contains a full anisotropic description *U* *^ij^* for each atom. In each panel the condition in (4) is satisfied, indicating that the fully anisotropic model is statistically justified, only where the yellow surface is above the blue surface. (*a*) At 1.3 Å resolution the fully anisotropic model is clearly justified. (*b*) At 1.5 Å resolution the test is not conclusive, although it indicates that the hybrid model is preferable for most possible values of the effective restraint weights. (*c*) At 1.7 Å resolution the fully anisotropic model can be justified only under the very unlikely hypothesis that the effective restraint weights are so strong as to act as constraints.

If true atomic resolution data have been measured, it is both justifiable and informative to refine a structural model con­taining anisotropic ADPs *U^ij^* for each atom (Schneider, 1996 ▶; Howard *et al.*, 2004 ▶). As the available resolution falls off from this extreme, the number of observations eventually becomes insufficient to support such a complex model and simpler alternative models should be considered. It is instructive to see whether the *R*-factor ratio test is capable of indicating this resolution-dependent breakdown in the validity of a fully anisotropic model. One way to explore this is to conduct a set of parallel refinements that use the same starting model and differ only in the resolution of the data used. Fig. 4 ▶ shows the result of three such parallel refinements using as a test case human carbonic anhydrase II. When data to 1.3 Å resolution are used (Fig. 4 ▶ *a*), the Hamilton test clearly indicates that it is justified to select a fully anisotropic model rather than a simpler hybrid model. If the data are limited to 1.7 Å resolution (Fig. 4 ▶ *c*), the same test clearly indicates that the fully anisotropic model is not justified, and thus the simpler hybrid model is preferable. Given the weak bounds we are able to place on *w*_geom_ and *w*_ADP_, it is perhaps not surprising that the analysis is indecisive at the intermediate resolution of 1.5 Å (Fig. 4 ▶ *b*). Over most of the range of the effective restraint weights in this intermediate case the *R*-­factor ratio test indicates we should reject use of the fully anisotropic model, but rejection is not indicated if *w*_ADP_ lies near its upper bound.

One could of course consider choosing a purely isotropic model even at very high resolution. Continuing with the use of carbonic anhydrase as a test case, Table 3 ▶ lists the outcomes of refining isotropic, hybrid and anisotropic models against 1.5 Å resolution data. Comparison of the fully isotropic model to the fully anisotropic model using the *R*-factor ratio test at this resolution yields an inconclusive result similar to that in Fig. 4 ▶(*b*). However, applying the *R*-factor ratio test to directly compare the purely isotropic model with the hybrid model clearly indicates that the hybrid model is preferred (not shown).

## 5. Experimental assessment of anisotropic models at various resolutions   

In cases where application of the Hamilton *R*-factor ratio test indicates that a more complex model should be rejected, can one find empirical evidence of defects in the rejected model? To address this question, we chose as a test case the well studied structure of human carbonic anhydrase II. Diffraction data for this structure are available to better than 0.90 Å resolution. We had previously refined atomic resolution models for this structure using several protocols (Behnke *et al.*, 2010 ▶). One of these was a 0.95 Å resolution refinement using *SHELXL* (Sheldrick & Schneider, 1997 ▶) that included full-matrix estimation of the final error in both the coordinates and the anisotropic ADP terms *U^ij^* (PDB entry 1lug). The 1lug model was chosen as a reference gold standard for assessing the accuracy of model ADPs obtained from refinement using data truncated to successively lower resolution limits. This is an idealized test case, as both the data and the starting model taken into refinement at lower resolutions are unrealistically good. That is, an atomic resolution data set truncated to, say, 1.8 Å is of better quality than a typical 1.8 Å resolution data set. Furthermore, the starting model taken into refinement included features identified in the original atomic resolution refinement, for example alternate conformations and partial-occupancy water sites, that would not typically be part of a model initially determined at lower resolution. For these reasons it is probable that this idealized test underestimates the typical degradation in the accuracy of model parameters at any specific resolution. Nevertheless, the statistical signatures of increasing model degradation as the data available for refinement decrease should parallel that expected for less ideal data.

**Figure 5:**
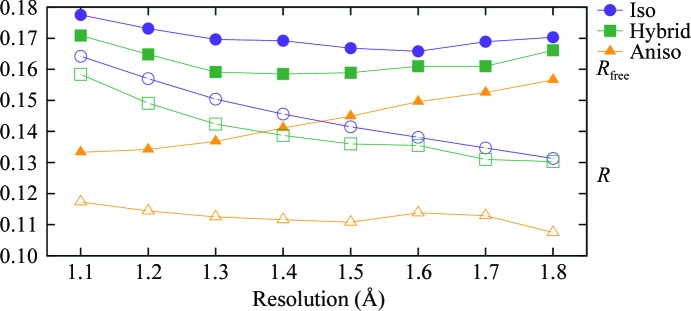
Refinement of human carbonic anhydrase II using atomic resolution data truncated to successively lower resolution. The plot shows the conventional crystallographic residuals *R* and *R*_free_ after refinement of the same starting coordinates using either an isotropic ADP *B*_iso_ for each atom, an anisotropic ADP tensor *U* *^ij^* for each atom or a hybrid model containing an isotropic ADP *B*_iso_ for each atom in addition to 16 TLS groups. All refinements started from the same set of positional coordinates and isotropic ADPs.

Fig. 5 ▶ shows the conventional crystallographic residuals *R* and *R*_free_ resulting from refinement of the same starting model using the 1lug 0.9 Å resolution data truncated successively to eight different resolution limits from 1.1 to 1.8 Å. At each resolution, three different models were refined, differing in their treatment of ADPs. The simplest, isotropic, model contained one ADP (*B*_iso_) for each atom. The most complex, fully anisotropic, model contained six ADPs (*U^ij^*) for each atom. The third model was a hybrid in which each atom was assigned an individual isotropic parameter *B*_iso_ and in addition the protein chain was divided into 16 segments each described by a set of 20 TLS parameters. The net anisotropic displacement of each atom in the hybrid model is thus the sum of contributions from the TLS description for the group to which it belongs and from the individual atomic *B*_iso_.

Note that at every resolution both *R* and *R*_free_ are highest for the isotropic model and lowest for the anisotropic model. Thus, if one were using only the existence of a drop in *R*_free_ as a guide to model selection the fully anisotropic model would be chosen even at the poorest resolution, 1.8 Å. As we saw in Fig. 4 ▶, this is contradicted by the Hamilton *R*-factor ratio test, which indicates that the decrease in *R* for the fully anisotropic model is not statistically significant for the lower resolution refinements and thus should be rejected. Because we have a gold standard available for comparison, we can directly assess the validity of ADPs obtained at lower resolutions by com­paring them atom-by-atom with the gold standard anisotropic ADPs in the atomic resolution 1lug model. We will use two statistical measures in this comparison: *S*_*UV*_ (Merritt, 1999*b* ▶) and the Kullback–Leibler divergence (Kullback & Leibler, 1951 ▶).

**Figure 6:**
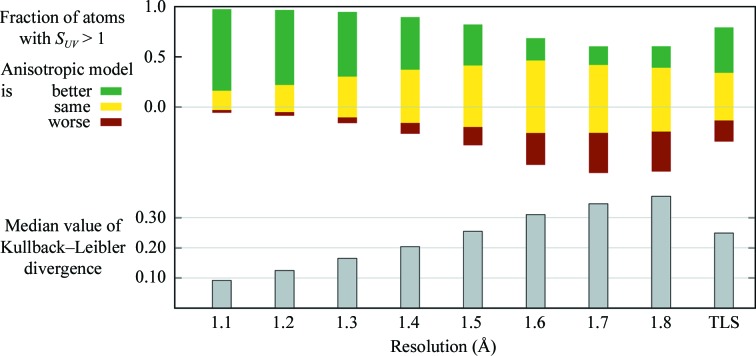
Experimental assessment of refining a fully anisotropic model at various resolutions. The ADPs deposited for the 0.95 Å resolution refinement of 1lug are used as a gold standard. The top set of bars shows the extent to which ADPs obtained from refining an anisotropic model against data truncated to successively lower resolution are a better approximation to the corresponding ‘true’ ADPs than would be obtained from a purely isotropic model. This comparison uses the statistic *S_UV_* (Merritt, 1999*b* ▶). The green portion of each bar corresponds to atoms with *S_UV_* > 1 + ∊, indicating that the electron density described by anisotropic treatment correlates better with that of the reference model than the density described by isotropic treatment. The red portion of each bar corresponds to atoms with *S_UV_* < 1 − ∊, indicating that anisotropic treatment yields a worse approximation to the reference density distribution than isotropic treatment. Values of *S_UV_* near 1.0 indicate that the anisotropic and isotropic models for that atom are equally good (or poor) approximations to the reference ellipsoid. The yellow portion of the bar is drawn for ∊ = 0.01. The lower set of bars show the extent to which the anisotropic ADPs obtained from refinement at truncated resolution diverge from those in the 0.95 Å reference model. If the model ADP *U* and the reference ADP *V* are identical, then the Kullback–Leibler divergence KL*_UV_* for that atom is equal to zero. Larger values of KL*_UV_* indicate increasing disparity between the electron-density distributions described by *U* and *V*. The height of each bar indicates the median value of KL*_UV_* calculated for all 2120 protein atoms in the refinement at that resolution. The rightmost bars show the same statistical assessments applied to a hybrid model containing 16 TLS groups in addition to a single parameter *B*_iso_ for each atom. The quality of the refined hybrid model is only weakly sensitive to truncation of the data in this resolution range; the bars shown are for refinement against data truncated to 1.5 Å resolution.

A symmetric form of the Kullback–Leibler divergence between the three-dimensional Gaussian density distributions described by *U* and *V* can be calculated using the equation KL*_UV_* = trace(*UV* ^−1^ + *VU* ^−1^ − 2*I*) (Murshudov *et al.*, 2011 ▶). In the present case, *U* is the tensor of gold-standard ADPs for a particular atom and *V* is a lower resolution anisotropic model for that same atom. The value of KL*_UV_* is zero if *U* = *V* and increases without bound as the difference between the two distributions increases. The lower set of bars in Fig. 6 ▶ shows the median value of KL*_UV_* obtained by comparing the ADPs *V* for every atom in each resolution-limited refinement with the gold-standard ADPs *U* for that same atom in the gold standard. This test shows that the refined ADPs in the resolution-limited anisotropic model refinements become increasingly divergent from the gold standard as the resolution limit becomes more severe. Although the numerical value of the Kullback–Leibler divergence does not by itself tell us at what resolution the model has diverged ‘too far’ from the gold standard, it does allow us to test at what point the ADPs obtained by refinement of a fully anisotropic model become worse than those obtained by refinement of a hybrid TLS model. As seen in Fig. 6 ▶, the ADPs from the hybrid TLS model are closer to the gold standard than the ADPs from the fully anisotropic model starting at 1.5 Å resolution.

The statistic *S_UV_* is based on the real-space correlation co­efficient between two electron-density distributions described by the pair of ADP tensors *U* and *V*. A value of *S_UV_* > 1 indicates that the electron-density distribution described by *U* correlates better with the anisotropic distribution described by *V* than it does with an isotropic distribution. A value of *S_UV_* < 1 indicates that the anisotropic model *V* has worse correlation than an isotropic model. Values of *S_UV_* very near to 1 indicate that the agreement of the isotropic and anisotropic models with the gold standard is approximately the same. The fraction of protein atoms in each of these categories is shown in the upper set of bars in Fig. 6 ▶.

As one would expect, full anisotropic refinement against data minimally truncated from 0.9 to 1.1 Å resolution does not sub­stantially reduce the agreement of the refined ADP values with the gold standard. Anisotropic treatment at this resolution is better than isotropic treatment for about 81% of the atoms and is no worse for another 17%. The quality of the refined ADP values degrades as the data are further truncated. By 1.6 Å, only 22% of the atoms are described better by an anisotropic model than by an isotropic model, and at this resolution the refined anisotropic ADPs for 32% of the atoms are actually a worse model for the true atomic resolution structure than an isotropic model. We can again compare this with similar analysis of refinement using a hybrid TLS model. In concordance with the analysis based on Kullback–Leibler divergence, the agreement of the hybrid model with the gold standard matches or exceeds that of the fully anisotropic model starting with the 1.5 Å resolution-limited refinement (Fig. 6 ▶).

Thus, evaluation of the refined models using either of two measures, *S_UV_* or Kullback–Leibler divergence, illustrates that as the resolution decreases the ADPs yielded by fully anisotropic refinement become invalid even though the refinement may remain numerically stable and the internal model statistics appear acceptable. This resolution-dependent breakdown in validity could not be detected by inspection of *R*_free_, which is lower for the fully anisotropic model than for either the isotropic or hybrid TLS models across the entire resolution range examined (1.1–1.8 Å). In contrast to this, the Hamilton *R*-factor ratio test is consistent with both empirical assessments in indicating that for this idealized test case the choice of a fully anisotropic model ceases to be justified at roughly 1.5 Å resolution.

## 6. Refinement protocols   

The model statistics in Tables 1 ▶, 2 ▶ and 3 ▶ are the result of refinement using *REFMAC* v.5.6.0095 (Murshudov *et al.*, 2011 ▶). In all cases the starting point for model comparisons was generated by subjecting the corresponding PDB entry coordinates to automated refinement in *REFMAC* with an iso­tropic *B* factor for each atom, no TLS treatment and default settings for all restraint weights. This coordinate set was then used as input for parallel refinements using alternative treatments for atomic displacements. Each refinement protocol was run first using a fixed overall geometric weighting term set to the value used in generating the starting model. If necessary, the refinement was then re-run using a manually adjusted value for the overall geometric weighting term chosen to yield deviations from ideality of the bond lengths and angles of the final model for that refinement protocol close to those of the starting model. Refinement of 3hzr used strong NCS restraints relating the six independent chains. Refinement of 3m5w used no NCS restraints. The refinements of 1lug in Table 3 ▶ were all conducted against data truncated to 1.5 Å resolution.

The parallel refinements of 1lug shown in Figs. 5 ▶ and 6 ▶ all used as a starting point the coordinates and isotropic ADPs from the 1.5 Å isotropic model shown in Table 3 ▶. The hybrid models additionally included a 16-group TLS model whose initial parameter values were taken from the 1.5 Å hybrid model shown in Table 3 ▶. In each case refinement consisted of 15 cycles of positional and ADP refinement; for the hybrid models, this was preceded by 15 cycles of TLS refinement. For anisotropic refinements, the along-bond ADP restraint RBON was set to 0.1. *REFMAC* was allowed to set the overall geo­metric weighting term automatically. The control settings for the individual refinements within a protocol (isotropic, hybrid, anisotropic) differed only in the resolution limit of the data used in refinement.

## 7. Concluding remarks: to *B* or not to *B*?   

Hamlet tempered his initial resolve by thinking about the significance of the alternatives available to him. My hope is that the examples presented here will encourage crystallo­graphers to do likewise. Before final acceptance of a structural model, even one that has been refined and validated, it is good to consider whether a simpler alternative model is available. Although the current discussion focuses on alternative treatments of *B* factors, this advice also applies to other model choices such as the treatment of non­crystallographic symmetry.

I have also taken the opportunity to explore the use of the largely neglected Hamilton *R*-factor ratio test as one approach to judging whether a more complex structural model is statistically justified. Widespread adoption of this test faces two hurdles: the key residual *R*_G_ is not reported by commonly used refinement programs and the test itself is weakened by the lack of a precise estimate for the effective restraint weights. The first hurdle can be easily overcome. For example, the PDB_REDO project is implementing automated evaluation of the Hamilton *R*-factor ratio for model selection (Joosten *et al.*, 2012 ▶). It may also be possible to lower the second hurdle by extending the argument advanced above to set weak bounds on *w*_ADP_ and *w*_geom_ so that it yields tighter bounds.

The choice ‘to *B*’ or ‘not to *B*’ is brought into sharp focus at both high and low resolution by the availability of TLS as an alternative description of atomic displacements. At high resolution the difference in number of parameters between a full anisotropic model and a hybrid *B*_iso_ + TLS model is more than a factor of two. At low resolution the difference in number of parameters between a model with individual *B*_iso_ terms and a pure TLS model is even larger. As we saw for the case of 3hzr in Figs. 2 ▶ and 3 ▶, the answer at 3 Å resolution is sometimes ‘not to *B*’.

Similarly, two different empirical assessments of ADP model quality using an atomic resolution structure determination as a gold standard illustrate that a drop in *R*_free_ is not a sufficient indication for choosing the more complex full anisotropic model at high resolution. The Hamilton *R*-factor ratio test, however, correctly indicates for the test case examined that a fully anisotropic model ceases to be valid at roughly 1.5 Å resolution. This particular resolution should not be interpreted as a new rule-of-thumb! Both the data quality and the starting model used in the idealized test case were unrealistically good for their nominal resolution. It seems likely that for a more representative starting model refined against more typical experimental data, the critical resolution at which a hybrid *B*_iso_ + TLS model becomes preferred to a full anisotropic model will lie closer to atomic resolution. In any case, the analysis summarized in Figs. 4 ▶, 5 ▶ and 6 ▶ reinforces the recommendation that statistical validation is desirable before accepting a model with a hugely larger number of parameters, even if it yields a decrease in *R*_free_.

## Figures and Tables

**Table 1:** Alternative ADP treatments of 3hzr at 3.0 resolution

	*U*_overall_	TLS only	*B*_iso_ only	TLS + *B*_iso_
*N*_reflections_ (working)	51642	51642	51642	51642
*N*_reflections_ (free)	2753	2753	2753	2753
*N*_parameters_	53202	53310	70928	71042
*N*_geometric_restraints_	176760	176760	176760	176760
*N*_ADP_restraints_	0	0	116074	116074
*R*/*R*_free_	0.2926/0.3097	0.2274/0.2455	0.2280/0.2716	0.2107/0.2399
*R*_G_/*R*_Gfree_	0.2827/0.2864	0.2366/0.2483	0.2283/0.2603	0.2248/0.2435

**Table 2:** Alternative ADP treatments of 3m5w at 2.32 resolution

	TLS only	*B*_iso_ only	TLS + *B*_iso_
*N*_reflections_ (working)	27045	27045	27045
*N*_reflections_ (free)	1997	1997	1997
*N*_parameters_	16033	21324	21364
*N*_geometric_restraints_	42540	42540	42540
*N*_ADP_restraints_	0	25199	25199
*R*/*R*_free_	0.2108/0.2723	0.1637/0.2319	0.1616/0.2265
*R*_G_/*R*_Gfree_	0.2532/0.3285	0.1989/0.2794	0.1969/0.2764

**Table 3:** Alternative ADP treatments of 1lug at 1.50 resolution

	*B*_iso_	*B*_iso_ + 1 TLS	*B*_iso_ + 16 TLS	*U^ij^*
*N*_reflections_ (working)	35493	35493	35493	35493
*N*_reflections_ (free)	1861	1861	1861	1861
*N*_parameters_	10064	10084	10384	22644
*N*_geometric_restraints_	18031	18055	18055	18025
*N*_ADP_restraints_	10843	10846	10846	27281
*R*/*R*_free_	0.1395/0.1655	0.1333/0.1571	0.1333/0.1565	0.1110/0.1442
*R*_G_/*R*_Gfree_	0.2037/0.2430	0.1931/0.2289	0.1931/0.2267	0.1588/0.2082
